# Femtosecond FBG Written through the Coating for Sensing Applications

**DOI:** 10.3390/s17112519

**Published:** 2017-11-02

**Authors:** Joé Habel, Tommy Boilard, Jean-Simon Frenière, François Trépanier, Martin Bernier

**Affiliations:** 1 Center for Optics, Photonics, and Lasers (COPL), Université Laval, Québec, QC G1K 7P4, Canada; tommy.boilard.1@ulaval.ca (T.B.); jean-simon.freniere.1@ulaval.ca (J.-S.F.); Martin.Bernier@copl.ulaval.ca (M.B.); 2 TeraXion Inc., Québec, QC G1P 4S8, Canada; ftrepanier@teraxion.com

**Keywords:** Fiber Bragg gratings, fiber sensors, ultrafast material processing

## Abstract

Type I fiber Bragg gratings (FBG) written through the coating of various off-the-shelf silica fibers with a femtosecond laser and the phase-mask technique are reported. Inscription through most of the common coating compositions (acrylate, silicone and polyimide) is reported as well as writing through the polyimide coating of various fiber cladding diameters, down to 50 µm. The long term annealing behavior of type I gratings written in a pure silica core fiber is also reported as well as a comparison of the mechanical resistance of type I and II FBG. The high mechanical resistance of the resulting type I FBG is shown to be useful for the fabrication of various distributed FBG arrays written using a single period phase-mask. The strain sensing response of such distributed arrays is also presented.

## 1. Introduction

Fiber Bragg grating (FBG) has been an active research domain for the last 40 years [1]. Sensor applications of FBG were first proposed 30 years ago [2] and continue nowadays to be an active research field [3]. FBG writing can be achieved with UV radiation [4,5,6] or with infrared (IR) femtosecond (fs) pulses [7,8]. The index change related to type I FBG is generally resulting from a combination of glass densification and color centers formation [6], while type II FBG is resulting from a damage process in the glass matrix [9]. UV-induced modifications of the refractive index rely on single- and multi-photon absorption [5,6,10,11] while writing with IR fs pulses rely exclusively on a multi-photon absorption process [8,9]. Such process has some significant advantages for sensing applications as it makes writing in non-UV photosensitive glasses possible. For example, FBG writing has been reported in pure silica with both IR and UV light [8,12,13] but mainly with IR fs pulses for fluoride [14] and chalcogenide glass fibers [15]. The resulting IR-FBGs can also have higher temperature stability than their UV-FBG counterparts when type II FBGs are considered [8]. IR fs pulses also facilitate writing through the coating, as most polymers are IR transparent [16,17]. This simplifies the fabrication process, with no necessity to strip the fiber prior to laser exposition and to recoat the grating afterwards, which ultimately limit the fiber robustness and the FBG manufacturing reliability. The devices reported in this paper have all been written through the coating (WTC).

It is possible to write a UV-FBG through the coating. Starodubov et al. used a 351 nm source to reduce the coating absorption to 25% [18]. UV-transparent coatings have also been developed [19,20]. However, with UV irradiation, no significant index modulation has yet been achieved in standard silica fibers with their coating. Another way to make a FBG without uncoating the fiber is by writing directly on the draw tower before the coating process [21,22]. This method has many advantages in term of manufacturability, but it is limited to single-pulse exposure. This is an important limitation of the technique, as the number of pulses affects directly the maximum index modulation that can be achieve, which ultimately restrict significantly the achievable FBG design. 

In this paper, type I fs IR FBG written through various coating are reported. Our group showed recently the possibility to write a type I FBG through the acrylate and polyimide coating of unloaded silica fiber without any degradation of mechanical strength [23]. Loading the fiber with deuterium to reach large refractive index modulations, up to 5 × 10^−3^, and post-annealing such FBG to reduce its internal losses demonstrated recently promising perspectives for sensing at very high resolution [24]. This article now focuses on the potential of these FBGs with high-mechanical strength for sensing applications.

## 2. Materials and Methods 

**Figure 1:**
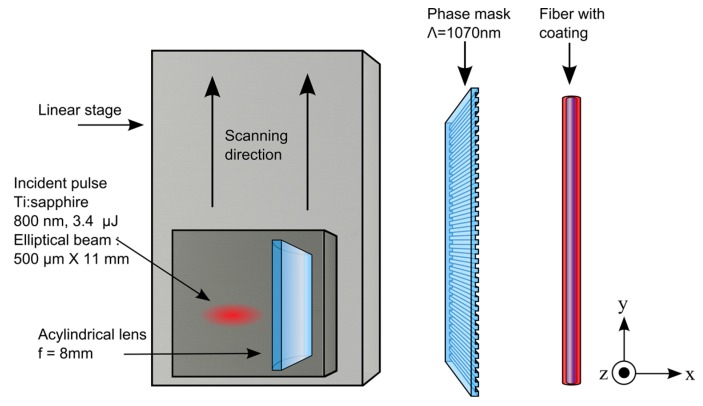
Fiber Bragg grating (FBG) writing set-up for an elliptical reshaped beam. Some results reported in this paper were obtained with a Gaussian beam (d ~ 11 mm) written on the same writing set-up.

**Figure 2:**
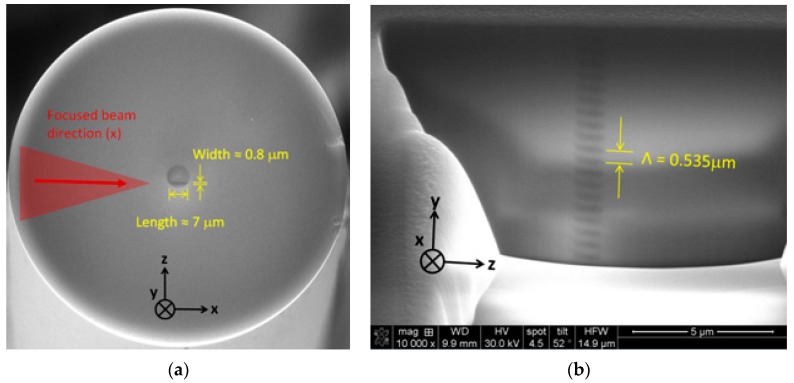
Scanning electron microscope (SEM) image of: (**a**) the cleaved fiber cross section; and (**b**) longitudinal view of the grating pattern, both for a static exposition of 60 s at 75 µJ of energy. The laser beam used was Gaussian, with d ~11 mm. The fiber is an unloaded SMF-28.

**Figure 3:**
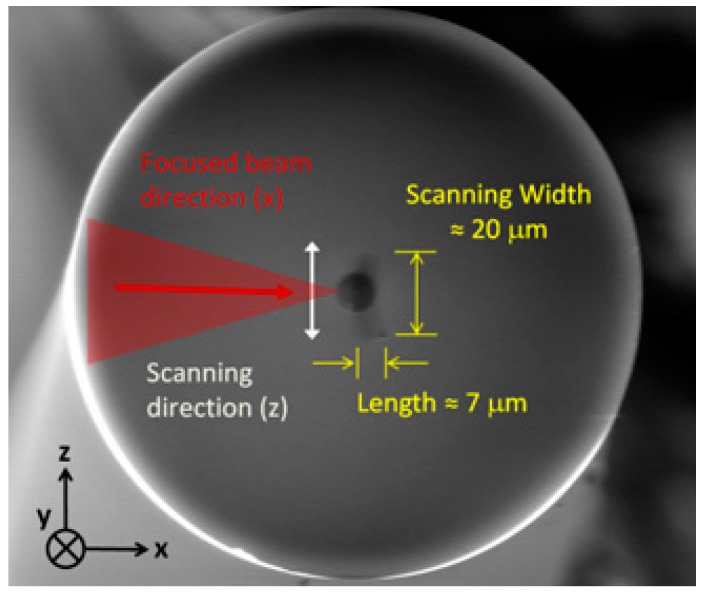
SEM image of the fiber cross section with transverse scanning over ±10 µm for an exposure time of 60 s at 75 µJ of energy. The laser beam used was Gaussian, with d ~11 mm. The fiber is an unloaded SMF-28.

The experimental phase-mask writing set-up is shown in Figure 1. A Ti:Sapphire regenerative amplifier system (Astrella, Coherent Inc., Santa Clara, CA, USA) that can produce up to 6 mJ pulses at 1 kHz with a central wavelength of 806 nm was used. The temporal width of the Fourier transform-limited pulses was measured to be ~ 30 fs. The 11 mm (1/e^2^) Gaussian output beam was used directly for some results presented in the paper and was reshaped to an elliptical 500 µm × 11 mm beam for other results when a control over the FBG length was targeted. The pulse energy is chosen for optimal type I writing condition, above the type I threshold and below the type II damage threshold. An acylindrical lens with a focal length of 8 mm focuses the beam through a phase-mask having a uniform pitch of 1070 nm producing a fundamental Bragg resonance around 1550 nm in standard silica fibers. The lens is mounted on a piezoelectric translation stage to scan the beam transversally (*z* direction, see Figure 1), over ±10 µm in order for the grating to better overlap the fiber core. Figure 2a shows a scanning electron microscope (SEM) image of the fiber cross section modified by laser exposure during a static exposition (without piezo scanning). The dark line in the bottom of the fiber core having a width of ≈0.8 µm and a length of ≈7 µm is the resulting modified region by static exposure. The fiber cross section was etched afterward along the *y* direction around the modified region by a focused ion beam etching system that equips the SEM (FEI, Quanta 3D-FEG). Figure 2b shows the FBG grating pattern along the *y* direction confirming the FBG pitch of 0.535 µm, half of the 1.07 µm phase-mask pitch, as expected. Figure 3 shows an SEM image of a fiber cross section after modification with a scanned exposure over ±10 µm. The width of the modified region is ≈20 µm, compared to 0.8 µm for a static exposition, with a curvature along the scanning axis that follow the fiber entrance interface curvature because of refraction. To precisely customize the FBG design length, the narrow 500 µm reshaped beam is scanned along the *y* direction at constant speed using a linear air bearing stage.

The ability to achieve the WTC process is highly dependent on the quality of the laser beam focusing geometry. The ratio between the laser intensity at the core and at the coating interface is a critical factor limiting the writing process and the resulting fiber mechanical robustness. An intensity ratio of ~100 is calculated using Zemax for a fiber with a cladding diameter of 125 µm and the 8 mm focal length acylindrical lens used for the experiment. 

## 3. Results

### 3.1. Mechanical Strength

**Figure 4:**
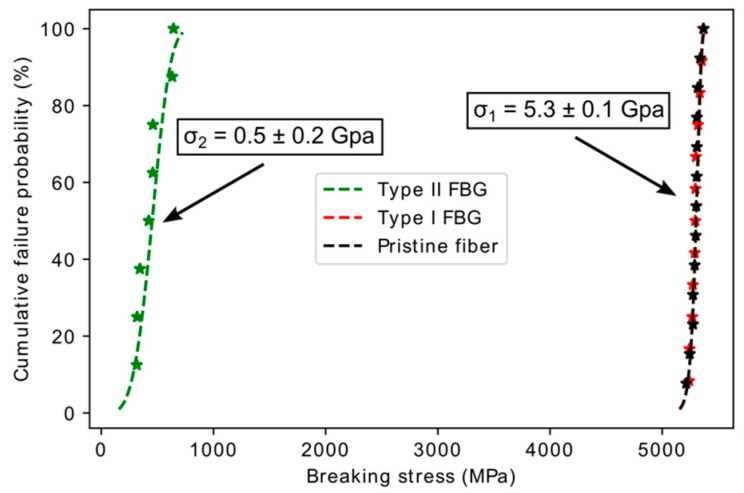
Comparison of the mechanical strength of pristine fiber versus type I and type II FBG written through the coating (WTC) of SMF-28 fiber. Results were partially reported in [23].

To emphasize the importance of writing type I gratings when mechanical reliability is targeted, we compare the mechanical strength of type I and type II FBG WTC. Eight type II gratings were written in SMF-28 fiber using the same experimental conditions as type I FBG but with a pulse energy well above the damage threshold (~200 µJ for type II vs. ~75 µJ for type I for a non-reshaped Gaussian laser beam of d ~11 mm). They were then pull-tested at a rate of 5% minimum using a build-in-house pull-tester. A Weibull plot was made from these breaking stresses values. In Figure 4, these new results are added to the type I and pristine Weibull plots for SMF-28 fiber from [23]. The pristine fiber and the type I gratings have a mean breaking stress of 5.3 GPa, ten-fold above the 0.5 GPa value obtained for type II FBG. This was expected as the type II gratings are defined by structural damages induced in the fiber. 

The type II gratings, at 0.5 GPa of mean breaking stress, has better mechanical strength than type I grating undergoing annealing treatment at 900 °C (mean breaking stress of 0.1 GPa [25]), which is the usual temperature for regenerated grating [25]. The type I FBG breaking stress value of 5.3 GPa is higher than a previously reported value of 4.6 GPa for a commercial type I FBG written in silica fibers [25]. However, in [25], the chemical composition of the fiber is different and the FBGs were uncoated for UV writing purpose. This may explain the observed difference.

### 3.2. High Temperature Limits of Type I FBG

**Figure 5:**
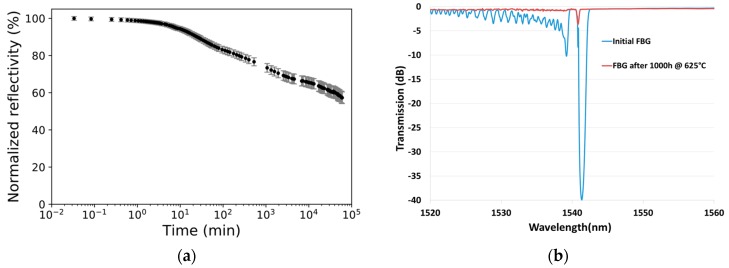
(**a**) Normalized refractive index change to initial strength of a type I FBG at 625 °C for 1000 h in a pure silica core fiber (Fibercore SM1500SC(9/125)P); and (**b**) its initial and final transmission spectrum.

To first investigate the high temperature performance of the fs type I FBG WTC, we monitor, for 1000 h, the annealing process of a saturated FBG written in a pure silica core fiber (Fibercore SM1500SC(9/125)P) written at ~90 µJ of pulse energy for 300 s exposure using a non-reshaped Gaussian laser beam of d ~11 mm. A specialized optical fiber oven (model ASP-500C, American Speciality Products Inc., Vernon, CT, USA) customized to maintain the FBG up to 750 °C in a controlled inert environment (ultra-high purity nitrogen) was used for the annealing process. A temperature of 625 °C was chosen for the experiment to keep the annealing temperature close to the densification stability limit [26]. Figure 5a shows the evolution of the normalized reflectivity for the resulting grating along the annealing time while Figure 5b shows the corresponding initial and final FBG transmission spectrum. We can observe a final normalized reflectivity slightly above 50%, of 57 ± 2%. The FBG spectra presented in Figure 5b were also modeled (OptiGrating, Optiwave Inc., Nepean, ON, Canada) by assuming a Gaussian apodization and sinusoidal fringe pattern. Only about 20% of the original induced refractive index change remains after this long annealing process. However, this grating is not stable and a longer exposure to 625 °C would reduce its reflectivity even more. The same effect would be expected in a shorter time scale if the temperature was raised above 625 °C. The usability of type I FBG above such temperature limit value is then compromised.

To operate at higher temperature, other alternatives were reported, such as regenerated gratings [27,28,29,30], type II gratings [31,32], or inscription in crystalline fibers that can sustain much higher temperature such as sapphire [33,34,35]. We should note that the polyimide coating deteriorated above 400 °C during this annealing process, while an acrylate coating deteriorates above 150 °C for a short term operation. For most off-the-shelf fibers, when the original coating needs to be preserved, the high temperature limit of the device is defined by the coating’s high temperature limit.

### 3.3. Writing through Various Coating Composition and Fiber Geometry

**Figure 6:**
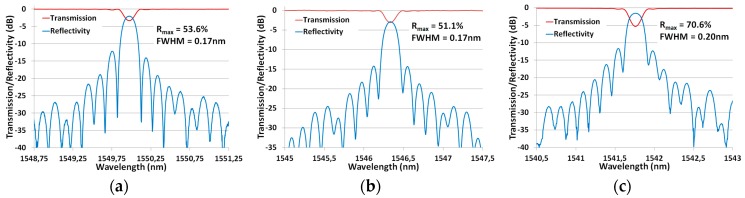
Transmission/reflectivity spectra of FBG written in fiber with: (**a**) High-temperature acrylate coating (Fibercore SM1500(9/125)HT); (**b**) silicone coating (Fibercore SM1500(9/125)S); and (**c**) polyimide coating with pure silica core (Fibercore SM1500SC(9/125)P).

A larger variety of coatings through which writing a FBG is possible offer more possibilities regarding the sensor’s design and performance. The targeted FBG design, adapted to sensing applications, is 6 mm long FBG with at least 40% reflectivity and a FWHM ≤ 0.20 nm. These specifications require a refractive index modulation of 1–2 × 10^−4^, a value easily achievable with fs type I FBG WTC [17]. We then implemented writing such FBG design using the writing setup shown in Figure 1 through various coating compositions. Figure 6a–c reports the transmission and reflectivity spectra of the resulting FBG with high-temperature acrylate (Fibercore SM1500(9/125)HT), silicone (Fibercore SM1500(9/125)S) and polyimide (Fibercore SM1500SC(9/125)P) coatings, respectively. The two first fibers had a germanosilicate core while the third one had a pure silica core. The pulse energy of 3.4 µJ (reshaped elliptical beam of 500 µm in length) was used for the germanosilicate fibers while it had to be raised to 4.0 µJ for the pure silica core fiber. The typical writing speed (*y* direction) was set to 5–10 mm/min, which means about 30–60 s exposure to reach the 5 mm targeted design length.

The writing speed was set to reach the targeted specifications but a stronger reflectivity could have easily been achieved by reducing the writing speed until saturation at a maximum index modulation of about 1 × 10^−3^ in unloaded standard silica fibers. The gratings exhibit negligible losses due to cladding modes coupling and over 10 dB of side mode suppression. These are important specifications for the FBG’s distributed sensing potential. 

In addition to the variety of coatings that can be written through, the ability to write FBG in smaller-sized fiber also extends the possibility for sensor’s design. This is of great practical interest as a smaller diameter makes the fiber more robust against bending, making it more adapted for smart sensing. 

**Figure 7:**
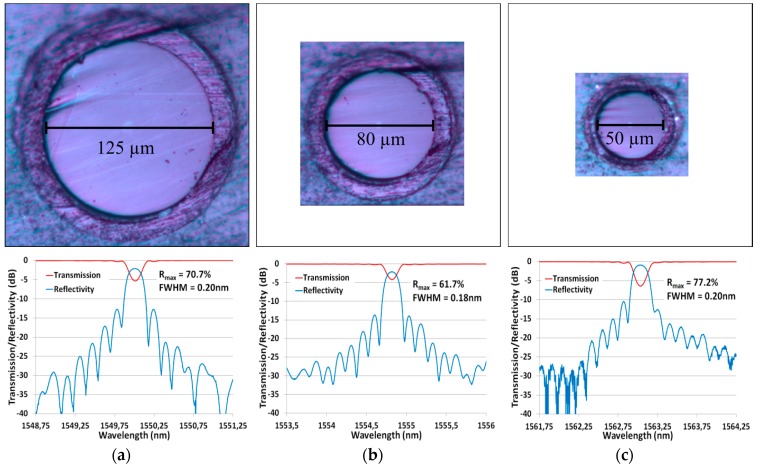
(**Top**) Optical microscope images of the polished cross-section for 125, 80 and 50 µm cladding diameter fibers; and (**Bottom**) their corresponding transmission and reflectivity spectra. Fibercore’s fibers are: (**a**) SM1500(9/125)P; (**b**)SM1500(5.1/80)P; and (**c**) SM1500(4.2/50)P. Note that (**c**) was D_2_-loaded prior to inscription to reach the targeted design.

We report the inscription of FBG in polyimide fibers (with d_core_/d_clad_= 9/125, 5.1/80 and 4.2/50 µm) with germanosilicate cores. An important challenge for writing FBG WTC in smaller fibers is related to the core-to-cladding intensity ratio as it limits the pulse energy that can be used without damaging the coating. These ratios are, respectively, ~100, 65 and 40 for fibers of 125, 80 and 50 µm cladding diameters. To achieve the required index modulation in the smallest fiber, deuterium-loading prior to the inscription was needed, as this process enhances the fiber’s photosensitivity. Figure 7 shows the polished fiber’s cross-section at the same imaging scale along with the resulting transmission and reflectivity spectra for FBG with the same targeted design detailed above.

The optical microscope images, presented in Figure 7 with consistent scales, highlight the importance of the focusing geometry when writing through the coating of a small fiber. The resulting FBG meets the specifications (FWHM ≤ 0.20 nm, R ≥ 40%) for the three fibers but the smallest fiber (SM1500(4.2/50)P) had to be deuterium-loaded to enhance its photosensitivity. The gratings are also not saturated, have no significant cladding mode losses and ~10 dB isolation.

### 3.4. Strain Testing

**Figure 8:**
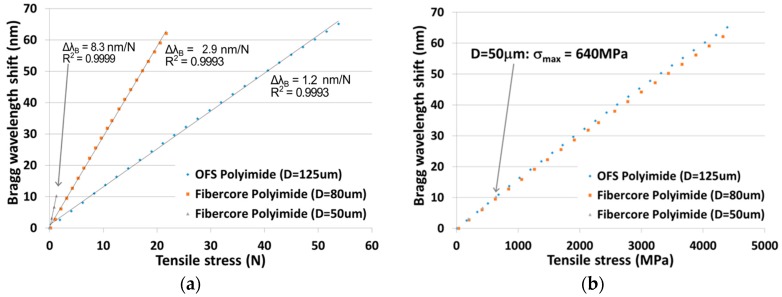
Bragg wavelength shift to tensile stress in: (**a**) N; and (**b**) MPa, for three different-sized polyimide fibers.

The gratings written in polyimide fibers (presented in Figure 7) were strain tested after fabrication. The shift of the Bragg wavelength was monitored according to the increasing strain, using our pull-tester. We can see on Figure 8a that, the smaller the fiber is, the more sensitive to loading it becomes, as expected. The sensitivities measured are 1.2, 2.9 and 8.3 nm/N. This flexible sensitivity, dependent on the fiber diameter, is another advantage of using smaller fibers for sensing. We can also notice the highly linear sensor’s response, with R^2^ ≥ 0.9993. Precisions on the Bragg wavelength shift and the tensile stress are respectively of 0.1 nm and 1%. 

Figure 8b reports the same results normalized to the fibers cross-section. The maximum stress that can be measured is 4500 MPa with both the 80 and the 125 µm fibers. It is then possible to measure strain up to ΔL/L ≈ 5.5% (55 000 µε) which correspond to a maximum Bragg wavelength shift of 65 nm around 1550 nm. We can also see that the mechanical strength of the smallest diameter fiber was affected by the writing process, as the fiber broke at only σ = 640 MPa. This shows that the coating has been damaged during the inscription process and that damaging a little the coating is catastrophic for the mechanical reliability of the device. More optimization of the writing set-up needs to be done to fabricate highly resistant FBG WTC in fibers smaller than 80 µm.

**Figure 9:**
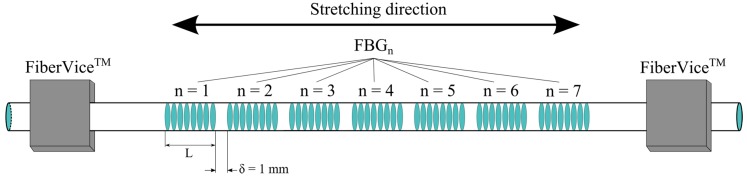
Experimental set-up for the strain testing of an array of FBGs.

**Figure 10:**
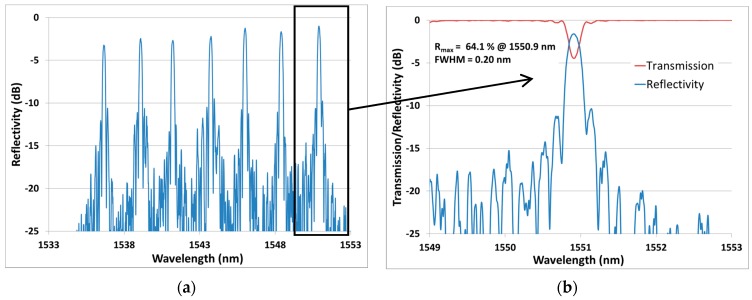
(**a**) Reflectivity spectrum of an array of seven FBGs (L = 5 mm) spectrally spaced by ~2.5 nm written in a Fibercore SM1500(9/125)P. (**b**) Zoom on the transmission and reflectivity spectra of one FBG.

To demonstrate the potential of this high mechanical strength FBG writing process, we performed the writing of an array of FBGs at different wavelengths using the same uniform phase-mask. Seven FBG have been written through the coating of a single unloaded polyimide fiber (Fibercore SM1500(9/125)P). The spacing between each grating was set to 1 mm, as shown in Figure 9. To control the Bragg wavelength of each FBG, we strained the fiber prior to exposure. The reliability of the fiber holders limits the maximum strain that can be applied to the fiber. We used FiberVice^TM^ grippers from PhotoNova Inc. A spectral spacing of 2.5 nm between each FBG was arbitrarily chosen. This value could easily be adjusted depending on the device’s application. A first array has been fabricated with a FBG length of 5 mm. Its transmission and reflectivity spectra are presented in Figure 10. 

**Figure 11:**
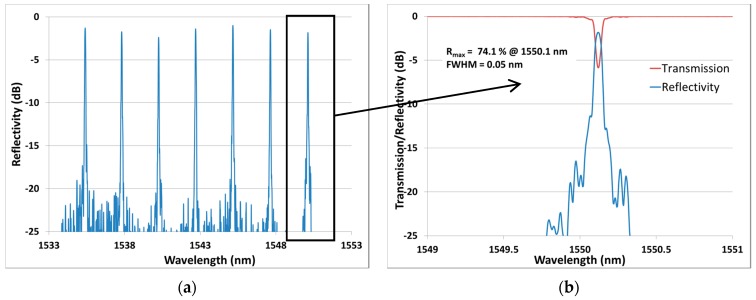
(**a**) Reflectivity spectrum of an array of seven FBGs (L = 25 mm) spectrally spaced by ~2.5 nm written in a Fibercore SM1500(9/125)P. (**b**) Zoom on the transmission and reflectivity spectra of one FBG.

**Figure 12:**
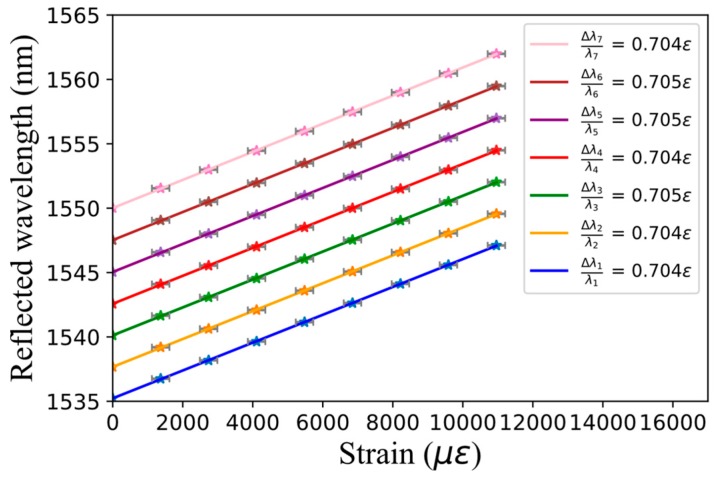
Reflected wavelength as a function of strain for all seven FBGs of the array presented in Figure 11.

The ability to precisely control the Bragg wavelength is important for distributed sensing. Figure 10a clearly shows how the spacing in the spectral domain makes it easy to track the FBG peaks. Each FBG in this array has R > 50% and FWHM ≤ 0.20 nm. To highlight the flexibility of the process, a second array with a longer FBG length of 25 mm was fabricated. It was then submitted to a strain testing experiment. The corresponding results are reported in Figure 11 and Figure 12.

Each FBG exhibits more than 50% reflectivity. We notice that the only significant change in the second array compared to the first one is the narrower spectral width of FWHM = 0.05 nm, as expected for such longer FBG length. This second array presented in Figure 11 has then been strain-tested up to 11,000 µε, as shown in Figure 12. We can see that a spectral spacing of 2.5 nm is once again obtained. Strong linearity and highly similar FBG sensitivities of 0.704 and 0.705 µε^−1^ are obtained for each grating. These sensitivities are slightly smaller than the theoretical value of 0.78 for silica [36]. Previous experimental sensitivities of 0.58 and 0.65 were obtained in [36,37]. These discrepancies are probably due to the difference in the fibers and coatings composition. If strain acts only on one FBG, the grating can measure a strain up to 2300 µε before overlapping the next grating. This limitation can easily be adapted to the sensor’s application by choosing a different spectral spacing.

## 4. Conclusions

We report type I FBG written through the coating of different sensors-optimized fibers. A high-temperature analysis shows that the grating in pure silica core survives 1000 h at a temperature of 625 °C with only 20% of its initial refractive index modulation remaining. FBG were successfully written through different fiber’s coatings and cladding diameters. Strain sensing showed a strong linearity, a sensitivity up to 8.3 nm/N and a maximum measured strain of ΔL/L ≈ 5.5% (55,000 µε) for different FBG. These results highlight the flexibility of the fs-IR FBG writing technique and its potential for sensing applications. The ability to write arrays of FBG through the coating highlights the potential of these devices for robust distributed sensing applications. These devices can now easily be adapted for mass production.
